# Case report: Diagnosis of visceral leishmaniasis using metagenomic next-generation sequencing and bone marrow smear

**DOI:** 10.3389/fcimb.2022.1095072

**Published:** 2022-12-23

**Authors:** Xiaogang Zhang, Yanqing Liu, Mingming Zhang, Zhiqiang Wang, Xing Feng, Liu Yang, Yajing Wang, Juan Liu, Dongbao Zhao

**Affiliations:** ^1^ Department of Rheumatology and Immunology, Changhai Hospital, Naval Medical University, Shanghai, China; ^2^ Department of Rheumatology and Immunology, The 980th Hospital of the Chinese People’s Liberation Army (PLA), Joint Logistics Support Force, Shijiazhuang, China

**Keywords:** visceral leishmaniasis, kala-azar, metagenomic next-generation sequencing, highthroughput sequencing, bone marrow smear, early diagnosis, China

## Abstract

Visceral leishmaniasis (VL) is a chronic infectious disease transmitted by sandflies. The primary clinical manifestations are remittent fever, pancytopenia, and splenomegaly. As VL is rare with atypical symptoms, its diagnosis is often incorrect, missed, or delayed. Without appropriate treatment, the case fatality rate of symptomatic disease is more than 95%, but the prognosis is good if diagnosed and treated timeously. We report a case of VL that was diagnosed using metagenomic next-generation sequencing (mNGS) of a peripheral blood sample. By using mNGS and a bone marrow smear, we were able to make a timely diagnosis. The patient was treated with antimony, rapidly recovered, and was discharged from the hospital. This case illustrates the value of mNGS for making a timely diagnosis of VL.

## 1 Introduction

Visceral leishmaniasis (VL), also known as kala-azar, is a chronic zoonotic infectious disease caused by *Leishmania* spp. Sandflies are the main natural transmission vectors. VL is endemic primarily in tropical and subtropical areas, with the highest incidence rates occurring in India, Bangladesh, Sudan, and Brazil *(* Ejazi and Ali, 2013; Scarpini et al., 2022 *)*. VL was eliminated in most of the formerly endemic areas in China almost 50 years ago *(* Dai et al., 2022 *)*. However, in recent years, sporadic cases have occurred, primarily in mountainous or remote rural areas in northwest China *(* Dai et al., 2022; Li et al., 2022 *)*.

The clinical symptoms of VL include fever, pancytopenia, splenomegaly, and emaciation. Patients may be asymptomatic in the initial stage of the disease, and the incubation period typically lasts several weeks to more than one year *(* van Griensven and Diro, 2019; Scarpini et al., 2022 *)*. The case fatality rate is more than 95% in individuals with symptomatic disease, if untreated or if treatment is delayed *(* Gao et al., 2022 *)*. As the disease is rare in China and its clinical symptoms are atypical, its diagnosis is often incorrect, missed, or delayed. However, the prognosis is good if diagnosed and treated timeously. Identification of the pathogen is the gold standard for diagnosis. However, limitations in spleen, bone marrow, and lymph node aspiration, include their invasiveness and the limited number of samples potentially obtained. Additionally, in many cases, the initial aspiration samples are negative, and patients may not consent to repeated sample collection. With advancements in molecular biology, metagenomic next-generation sequencing (mNGS) technology has been used to directly conduct high-throughput sequencing of patient samples. After obtaining the nucleic acid sequence information of pathogens, comparisons are made with database samples to learn the species of pathogenic microorganisms. This method has the advantages of speed, wide detection ranges, and high sensitivity and specificity. In this case, a peripheral blood sample from the patient was tested using mNGS. This procedure led to rapid identification of the pathogen and, combined with a bone marrow smear, led to a timely diagnosis. After standardized treatment, the patient rapidly recovered and was discharged. This case report provides an example of the value of mNGS for making a timely diagnosis of VL.

## 2 Case presentation

A 68-year-old man from a mountainous area in Hebei Province, China, presented to the Rheumatology and Immunization Department of The 980th Hospital in Shijiazhuang, Hebei Province on February 16, 2022 with fever, sweating, and fatigue. He had previously been hospitalized in Jingxing County Hospital of Traditional Chinese Medicine (TCM) in Shijiazhuang City, Hebei Province on February 1, 2022. On admission to Jingxing County Hospital of TCM, his temperature was 39.0°C. He had chills, but no cough, sputum, abdominal pain, diarrhea, or urinary symptoms. His blood test results showed pancytopenia, rapid erythrocyte sedimentation rate, elevated C-reactive protein and ferritin, hyponatremia, hypokalemia, hypoproteinemia, and elevated serum enzyme levels (**Table 1**). Bone marrow aspiration smear showed granulocytopenia with megakaryocyte maturation disorder. Abdominal ultrasound examination showed splenomegaly, a widened common bile duct, with no space-occupying lesions in liver, gallbladder, and kidney; brain computed tomography (CT) showed bilateral basal ganglia lacunar cerebral infarction; and lung CT showed bilateral lung fiber cord shadows, and bilateral increased lung branch air tube bundles. No definitive diagnosis was made during the previous hospitalization. After 11 days of antibiotic and symptomatic treatment, including administration of fluid and electrolytes, his symptoms persisted, he continued to have fever, fatigue, and a maximum temperature of >39°C.

**Table 1:** Data of laboratory examinations.

Items	Reference range	Previous hospital (Early Feb.)	Feb. 17	Feb. 18	Feb. 19	Feb. 20	Feb. 21	Feb. 22	Feb. 23	Feb. 24	Feb. 25	Feb. 26	Mar. 1	Mar. 3	Apr. 20
WBC (10^9^/L)	3.5–9.5	1.6	1.35	0.99	1.68	2.89	2.19	2.84	1.62	1.50	1.37	7.55	1.70	1.83	8.3
NEUT# (10^9^/L)	1.8–6.3		0.61	0.53	1.21	2.24	1.52	2.24	1.03	0.81	0.82	6.70	1.21	1.28	5.0
LYMPH# (10^9^/L)	1.1–3.2		0.63	0.36	0.36	0.51	0.55	0.45	0.45	0.54	0.40	0.60	0.33	0.33	1.9
RBC (T/L)	4.3–5.8		3.89	3.90	3.71	3.86	3.60	3.66	4.00	4.27	3.62	3.55	3.58	3.73	3.58
HGB (g/L)	130–175	90	92	94	89	92	87	88	96	102	88	86	89	93	96
PLT (10^9^/L)	125–350	38	21	18	38	26	26	29	36	38	48	61	104	141	196
UPRO			++											–	
K (mmol/L)	3.5–5.3		3.28		3.77				3.96			3.83			
Na (mmol/L)	137–147	126.5–132.6	126.6		131.3				132.4			134.1			
ALB (g/L)	40–55	23.3–25.9	22.2		29.9				29.6			28.6			
ALT (U/L)	9–50	64	65		58.1				49.5			31.0			
AST (U/L)	15–40		96.6		85.5				73.3			28.4			
ADA (U/L)	0-25		69.2		74.3				82.2			44.8			
LDH (U/L)	120–250		638		622				564			275			
HBDH (U/L)	72–182		411		403				359			212			
FER (ng/mL)	30–400	8,389.42	> 2,000									1,564.00			
ESR (mm/1h)	0–20	20	28									36			
CRP (mg/L)	< 10	52.95	57.00									5.690			
INR			1.38		1.21				1.13		1.08				
D-Dimer (mg/L)	0–0.243		11.697		7.507				11.609		0.999				
FBG (g/L)	2.38–4.98		1.57		1.62				1.33		1.44				

Other results of Lab. Examinations ANA: 1∶100 (< 1:100); ENA: Negative; RF (U/mL): 49.4 (0–20); anti-CCP (RU/mL) < 25 (0–25); C3 (g/L): 0.557 (0.7–1.4); C4 (g/L): 0.177 (0.1–0.4); PCT (ng/mL): 0.33 (0.02–0.5); PPD-C: Negative; CA199 (U/mL): 57.45 (≤ 30); CA50 (IU/mL): 51.45 (≤ 25); Gastrin-17 (pmol/L): 189.5 (1.7–7.6); 24-hour urinary protein (mg/24h): 992.2 (28–141); sCD25: 95,355pg/mL; NK cell activity of peripheral blood: 16.04%; Coombs test: Positive; EBV-DNA: Negative; HBVM: Negative; HCV-Ab: Negative; HCV-cAg: Negative; HIV-Ab: Negative; TP-Ab: Negative; Brucellosis antibody: Negative; Hemocultures: Negative.

**Figure 1:**
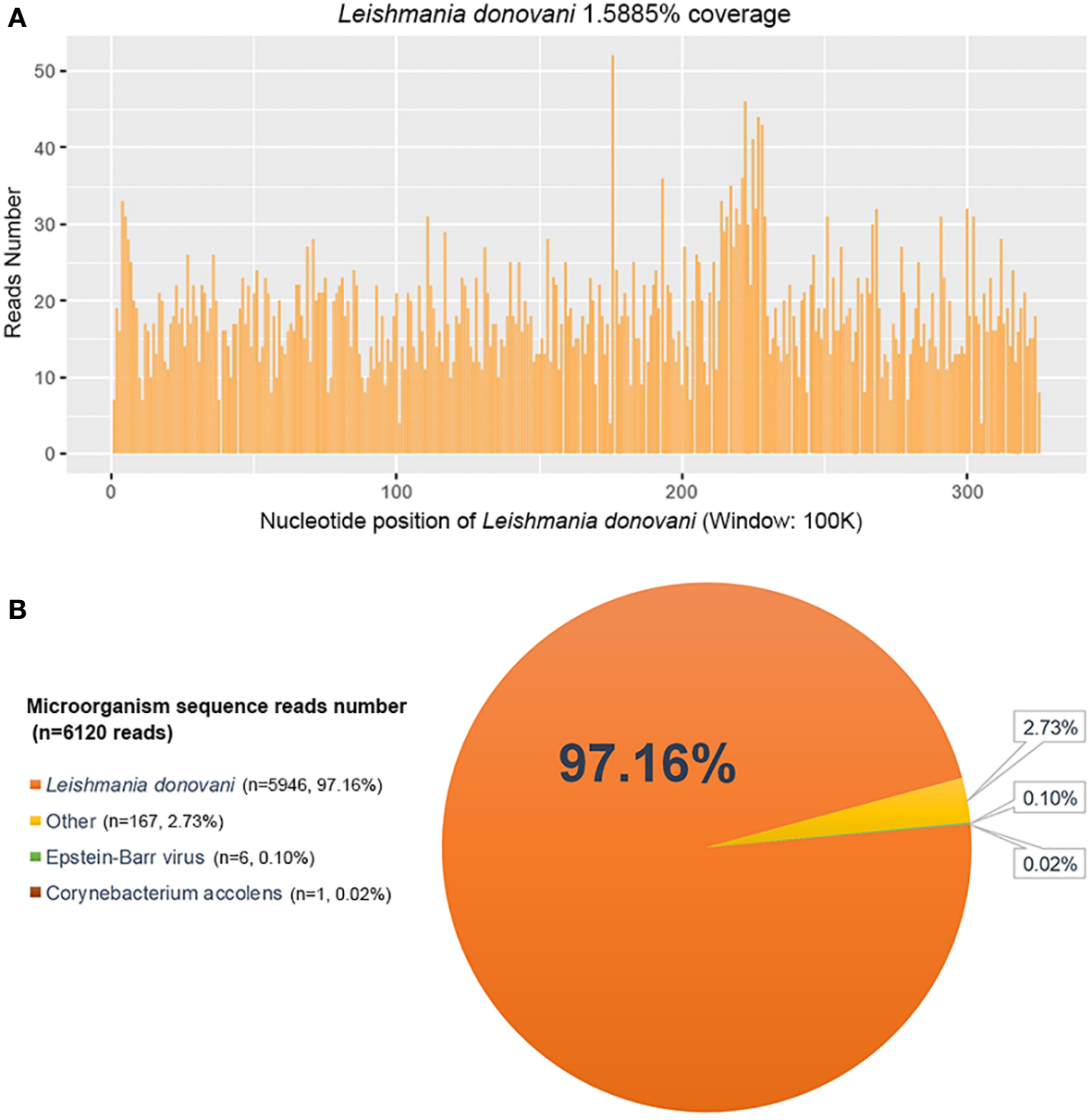
Metagenomic next-generation sequencing (mNGS) of peripheral blood sample of the patient. **(A)** The nucleotide position and coverage of *Leishmania donovani* detected by mNGS. The coverage was 1.5885%. **(B)** mNGS of blood samples showed a total of 6,120 reads identified as microbial genome sequences, of which 5,946 reads belonged to *Leishmania donovani*, accounting for 97.16% of the total.

The metagenomic next-generation sequencing (mNGS) (Feb. 21 2022) of blood samples showed a total of 6,120 reads identified as microorganism genome sequence, of which 5,946 (97.16%) reads belonged to *Leishmania donovani* The coverage rate of *Leishmania donovani* was 1.5885% (**Figure 1**).

**Figure 2:**
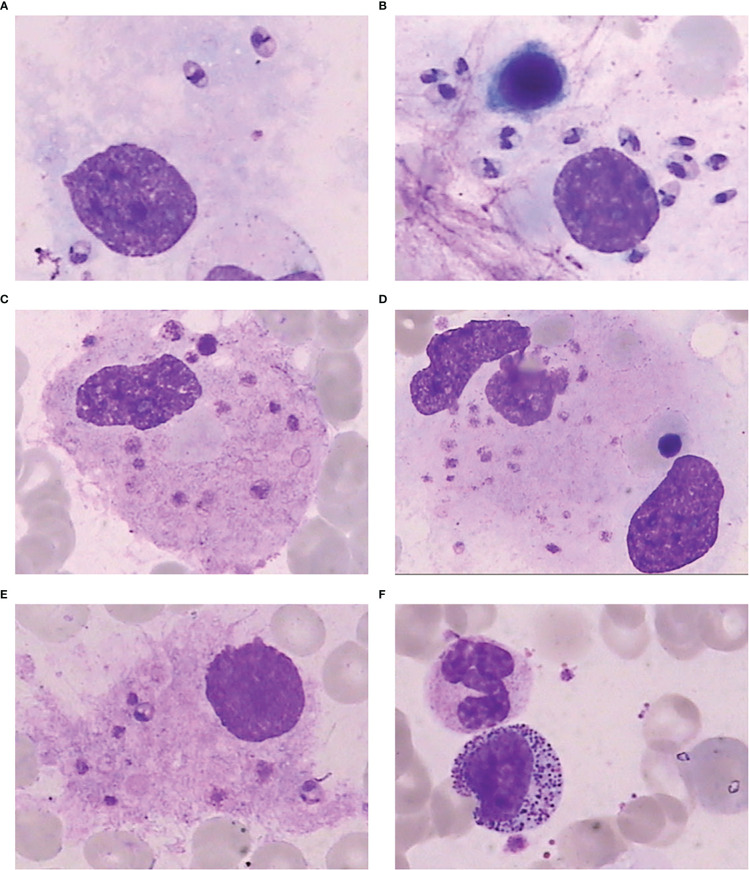
Bone marrow smear. **(A)** Amastigote in the bone marrow. **(B)** A large number of amastigotes in the bone marrow. **(C)** Phagocytosis of platelets by macrophages in the bone marrow. **(D)** Phagocytosis of platelets and nucleated red blood cells by macrophages in the bone marrow. **(E)** Amastigotes invading macrophages in the bone marrow. **(F)** Bone marrow smear after treatment showing normal morphology of bone marrow cells, with no hemophagocytic cells or amastigotes.

The bone marrow smear from the previous hospital (early Feb. 2022) showed granulopenia with megakaryocyte maturation disorder. The bone marrow smear (Feb. 21 2022) showed increased number of hemophagocytes, and amastigotes could been observed (**Figures 2A–E**). The bone marrow smear (Mar. 1 2022) showed no abnormalities in the morphology of bone marrow cells, no hemophagocytic cells, and no amastigotes (**Figure 2F**).

Abbreviations: ANA, antinuclear antibody; WBC, white blood cell; NEUT, neutrophil; LYMPH, lymphocyte; Hb, hemoglobin; PLT, platelet; UPRO, urinary protein; ALB, albumin; ALT, alanine aminotransferase; AST, aspartate transaminase; ADA, adenosine deaminase; LDH, lactate dehydrogenase; HBDH, α-hydroxybutyrate dehydrogenase; TG, triglyceride; FER, ferritin; ESR, erythrocyte sedimentation rate; CRP, C-reactive protein; RF, rheumatoid factor; FBG, Fibrinogen; HBVM, Serum hepatitis B markers; TP-Ab, treponema pallidum antibody; anti-CCP, anti-cyclic citrullinated peptide antibodies; PCT, procalcitonin, absolute count; +, positive.

He presented to our hospital for further diagnostic evaluation and treatment on February 16, 2022. Outpatient laboratory testing showed a negative SARS-CoV-2 polymerase chain reaction (PCR) test result, and chest CT showed signs of mild chronic inflammation in both lungs, slightly enlarged mediastinal lymph nodes, calcification of aorta and coronary arteries, a pericardial effusion, calcification under the capsule of the right lobe of the liver, and splenomegaly. After excluding SARS-CoV-2 infection, he was admitted to the Rheumatology and Immunology Department.

The patient had had no history of oral ulcers, rashes, joint swelling, or pain. He reported a decrease in body weight of approximately 10 kg in the past 6 months. His medical history included a resection of benign prostatic hyperplasia in July 2020, and a laparoscopic right hernia repair 3 months prior to admission. The patient did not have any history of infectious diseases such as hepatitis and tuberculosis. He had been vaccinated against COVID-19; however, his other vaccination history was unknown. Additionally, he was a non-smoker, did not drink alcohol, and had no family history of heritable diseases.

Physical examination on admission showed a temperature of 38.2°C, poor nutritional status, and an inability to walk. Additionally, his muscle strength in both legs was grade 3, and he had pitting edema on the anterior of both tibias and the dorsa of the feet.

**Figure 3:**
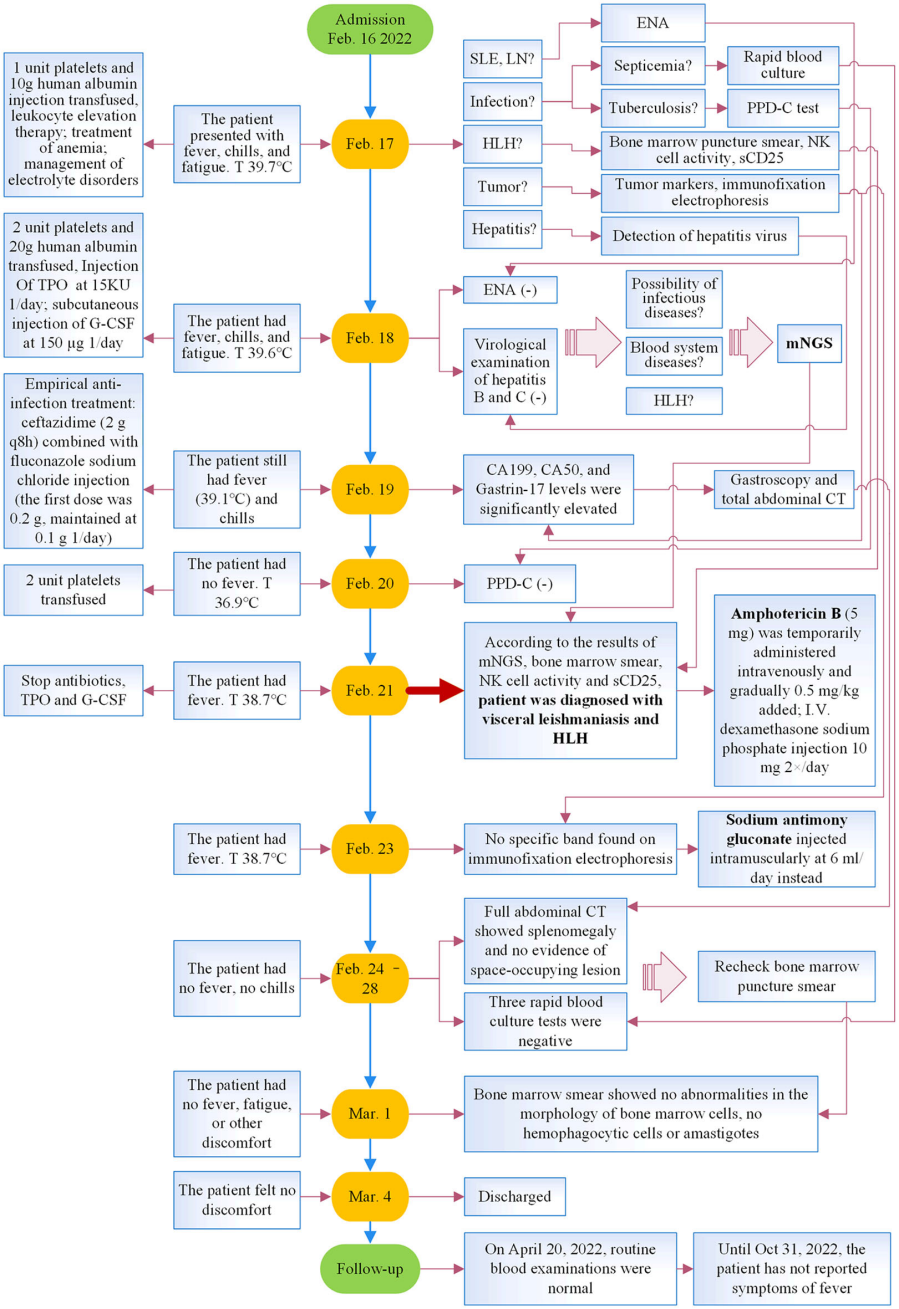
Timeline of the patient’s illness, diagnosis, and treatment. T, temperature; SLE, systemic lupus erythematosus; LN, lupus nephritis; ENA, autoantibody spectrum; HLH, hemophagocytic lymphohistiocytosis; TPO, recombinant human thrombopoietin; G-CSF, granulocyte colony-stimulating factor; mNGS, metagenomic next-generation sequencing; CT, computed tomography.

**Figure 4:**
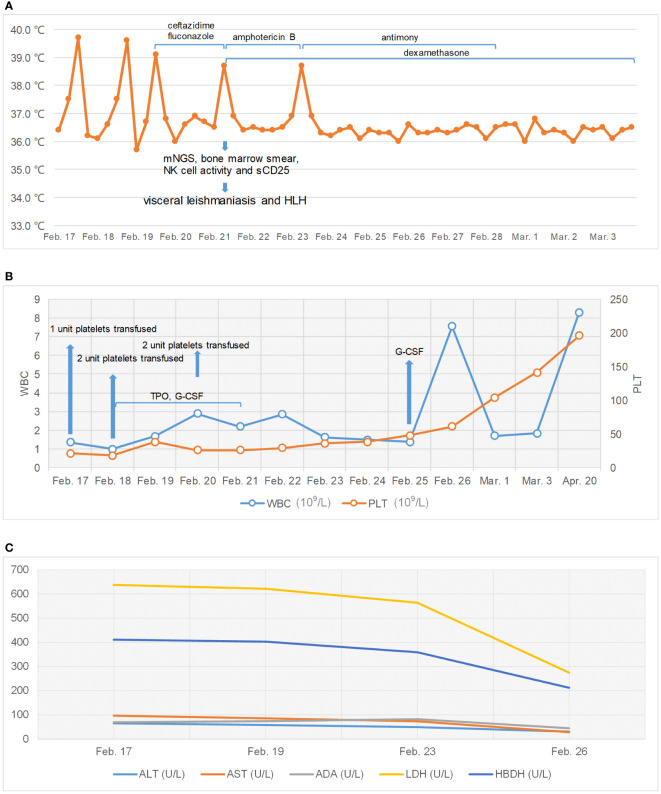
Changes of patient’s temperature, white blood cell (WBC), platelets (PLT), and serum enzyme levels. **(A)** Change of patient’s temperature. **(B)** Changes in WBC and PLT. **(C)** Changes in serum enzyme levels.

He underwent a series of laboratory tests after admission (**Table 1**). These tests revealed pancytopenia, increased ESR, CRP, and ferritin, and elevated liver enzymes. mNGS of blood samples showed a total of 6,120 reads identified as microbial genome sequences, of which 5,946 (97.16%) reads belonged to *Leishmania donovani* (**Figure 1**). Bone marrow smear revealed an increase in the number of hemophagocytes, and amastigotes could been observed (**Figures 2A–E**). Based on these results, he was diagnosed with VL and hemophagocytic lymphohistiocytosis (HLH), 5 days after admission. A timeline depicting the patient’s condition, diagnosis, and treatment is presented in **Figure 3**. After confirming the diagnosis of VL, anti-*leishmania* treatment should be administered as soon as possible. Antimony agent or amphotericin B is the commonly used drug, but only amphotericin B was available in the hospital pharmacy. To avoid further aggravation of the patient’s condition, amphotericin B 5mg/day was administered intravenously (gradually increased to 0.5mg/kg per day according to the patient’s tolerance), and dexamethasone sodium phosphate injection (10mg/day intravenous injection) was given for HLH induction treatment. Meanwhile, we considered the side effects of amphotericin B, which include fever, nausea, vomiting, and hypokalemia. With these side effects, the patient could not adhere to treatment, and it would not be conducive for efficacy evaluation. Therefore, after consultation with the patient and his family, we requested the hospital pharmacy to contact the antimony agent manufacturer for emergency drug delivery. The antimony agent was delivered two days later and replaced the amphotericin B immediately. A “six day plan” was adopted for antimony sodium gluconate (6ml 1/day intramuscular injection) for six consecutive days. The patient’s condition improved rapidly after treatment. The results of bone marrow puncture on March 1, 2022, showed no abnormalities in the morphology of bone marrow cells, and no hemophagocytic cells nor amastigotes were observed (**Figure 2F**). The changes in the patient’s laboratory indicators are shown in **Figure 4**. The patient was discharged from the hospital on March 4, 2022. After discharge, he underwent regular follow-up and remained in a stable condition for the following 8 months.

## 3 Discussion

Leishmaniasis is a chronic infectious disease that is transmitted in nature by sandflies. The disease is prevalent in tropical and subtropical regions, affecting more than 88 countries worldwide. The condition is most prevalent in India, Bangladesh, Sudan, South Sudan, and Ethiopia, where cases are caused primarily by *Leishmania donovani*, and in Brazil, where *Leishmania infantum* is prevalent. These six countries account for more than 90% of all VL cases *(* Ejazi and Ali, 2013; Zijlstra, 2019; Scarpini et al., 2022 *)*. In the early 1950s, leishmaniasis was one of the most serious parasitic diseases in China. By 1958, the disease had been eliminated nationwide. However, in recent years, sporadic cases have occurred in former endemic areas, including Xinjiang, Gansu, Shaanxi, and Shanxi *(* Zheng et al., 2020; Dai et al., 2022; Li et al., 2022 *)*.

There are three main clinical types of leishmaniasis: visceral, cutaneous, and mucosal *(* Scarpini et al., 2022 *)*. Cutaneous leishmaniasis is relatively rare in China, whereas VL occurs sporadically. The main pathogens of VL in China are *L. donovani* and *L. infantum*. In addition, within months to years after successful treatment, 10–60% of patients with VL may progressed into post-kala-azar dermal leishmaniasis (PKDL), mainly in *L. donovani* endemic areas (Indian subcontinent and East Africa) *(* Ejazi and Ali, 2013 *)*. Humans and dogs are the most important reservoir hosts; however, cats and rodents may also become potential hosts of of the pathogen *(* Quinnell and Courtenay, 2009; Mohebali et al., 2018; Nascimento et al., 2022 *)*.

After the sandfly is infected with *Leishmania*, the preflagellate rapidly propagates in its digestive tract and accumulates in large numbers in the oral cavity. When the sandfly bites a human, the preflagellate parasite (promastigote) enters the human body, infects peripheral macrophages, and grows into an oval non-flagellate body (amastigote) in the cells. After the macrophages rupture, the infection spreads to the liver, spleen, lymph nodes, bone marrow, and other internal organs, causing the body to produce a series of immune responses and resulting in symptoms *(* Ejazi and Ali, 2013 *)*.

After *leishmania* infects the human body, there may be no symptoms or signs at the early stages of infection. Asymptomatic infected individuals can transmit infection indirectly by transmitting it to sandflies through bites and the sandflies subsequently biting uninfected individuals, or directly through blood transfusion. Notably, screening of blood prior to transfusion does not include screening for *Leishmania* as is routinely undertaken for syphilis and HIV. Symptom onset in carriers is typically insidious and progresses slowly, with clinical manifestations that primarily include chronic irregular fever, emaciation, hepatosplenomegaly, anemia and thrombocytopenia or pancytopenia, and hypoproteinemia with hyperglobulinemia. The most serious and potentially fatal complications of VL are disseminated intravascular coagulation and HLH *(* Skinner et al., 2019; de Santana Ferreira et al., 2021 *)*. HLH was the main clinical manifestation of the patient in our case. Due to the non-specific symptoms of VL, some patients may not be diagnosed for long periods and may visit different health facilities with fever of unknown origin. Our patient had fever, emaciation, and pancytopenia, but a diagnosis could not be made based on these clinical manifestations alone, as several infectious diseases (including brucellosis, and tuberculosis), and autoimmune diseases (including systemic lupus erythematosus and Sjogren’s syndrome), can have similar clinical manifestations. Diagnosing leishmaniasis based solely on clinical manifestations is difficult, and the condition is often misdiagnosed as an autoimmune disease. Therefore, finding evidence of *Leishmania* infection is a more direct and accurate diagnostic tool.

Laboratory diagnosis of VL includes pathogenic, immunological, and molecular biological investigations *(* Ejazi and Ali, 2013 *)*. Identifying the pathogen, the gold standard for diagnosis, involve observing protozoan infections in smears of bone marrow, spleen, lymph nodes, and other organs and tissues, under a microscope. However, in the early stages of the disease, the protozoa may not have spread to tissues and organs, the number of protozoa in tissues and organs may be relatively small, and the predominant organs infected vary according to the geographic area. For example, in Brazil, protozoa often invade the bone marrow, rather than the spleen *(* Berman, 2006 *)*. However, tissue aspiration has limitations, including the invasiveness of the procedure and the small amount of sample obtained. In particular, with splenic aspiration, the risk of puncture is high, which may cause perforation and lead to death. Biopsies, when necessary, require multiple punctures to obtain tissue, which may result in physical and mental harm to patients, making it difficult to obtain consent for performing multiple procedures in patients with non-specific clinical manifestations in whom the probable diagnosis is unclear. In addition, analyzing smears under a microscope requires that clinicians to have extensive clinical experience. Therefore, biopsy may not be an effective method of diagnosis. However, bone marrow aspiration remains a common diagnostic procedure in conditions such as fever of unknown origin and hematologic diseases, and is important in the diagnosis of VL. Bone marrow aspiration in VL has low sensitivity and high specificity, which may also be affected by the ability and level of experience of the examiner. Prior to early February 2022, the bone marrow aspiration did not show amastigotes or hemophagocytosis, while on February 21, a large number of amastigotes and obvious hemophagocytosis were seen in the bone marrow smear conducted at our hospital. As the gold standard for diagnosis, bone marrow aspiration may be used to diagnose VL and is relatively safe compared with splenic puncture, so may be better accepted by patients.

Immunological detection also plays an important role in clinical practice, and using an enzyme-linked immunosorbent assay (ELISA) to detect the immunoglobulin G (IgG) antibody of rK39 is an important method for diagnosing leishmaniasis. However, a common disadvantage of antibody detection is that antibody tests may remain positive for a long period, even after successful treatment. A study that used ELISA to detect rK39 antibodies *(* Gidwani et al., 2011 *)* showed that 86.3% of patients with cured VL were still Ig G positive within one year of treatment, and that some patients were still positive 15 years later. It is not possible to determine whether the patient has active infection using antibody tests. Therefore, a positive rK39 test without clinical symptoms or other laboratory evidence is insufficient for diagnosis. Owing to its simplicity, low price, and rapidity, *Leishmania* antigen latex agglutination test (KAtex) of urine samples is also often used to screen for VL in countries or regions with limited resources. The sensitivity (36–94%) and specificity (64–100%) of KAtex are very variable *(* Alam et al., 2008; Ahsan et al., 2010; Vogt et al., 2018 *)*. However, the incidence of HIV-associated VL in Ethiopia, Brazil, and India is relatively high and increasing, and the use of KAtex may be beneficial, as antibody production is suppressed in HIV-infected individuals, so antibody-based testing has poor sensitivity, and KAtex is noninvasive *(* van Griensven et al., 2018 *)*.

Moreover, molecular biological tests have been increasingly used in the diagnosis of VL. PCR tests have high sensitivity (>95%) in spleen, bone marrow, lymph node, and peripheral blood samples. However, the specificity is low, and false-positive results may occur *(* van Griensven and Diro, 2019; Scarpini et al., 2022 *)*. In recent years, some studies have found that qPCR analysis of mitochondrial DNA minicircle network (kDNA) based on the characteristics of *Leishmania* genus has high sensitivity and specificity, and can thus be useful for quantitative detection of pan-*Leishmania (* Ceccarelli et al., 2020 *;* de Carvalho et al., 2022 *;* Mota et al., 2022 *)*. However, this technique is expensive and has not been used for routine clinical testing. mNGS is a molecular biological detection method that directly carries out high-throughput sequencing on clinical samples, obtains the nucleic acid sequence information of pathogens, and then compares it with a database through bioinformatics methods to determine the types of pathogenic microorganisms contained in the samples. This method has the advantages of fast detection, a wide detection range, and high sensitivity and specificity. In our case, a peripheral blood sample from the patient was tested using mNGS, and the pathogen was rapidly identified. Combined with a bone marrow smear, we were able to diagnose the patient’s condition in a timely manner.

In China, the diagnosis of VL is based on the *Expert Consensus on Diagnosis and Treatment of Leishmania Infection in China* (The Editorial Committee of Chinese Journal of Infectious Diseases, 2017). The diagnostic criteria include four aspects: (1) epidemiological history: residents in the endemic area of leishmaniasis, or have a life and work history in the endemic area; (2) clinical manifestations: long term remittent fever, progressive enlargement of spleen, mild or moderate enlargement of liver, decreased white blood cell and (or) platelet count, anemia, epistaxis and gingival bleeding, sometimes accompanied by lymph node enlargement; (3) serological testing: RK39 antibody or *Leishmania* latex agglutination test positive; (4) identification of the organism: *Leishmania* found on microscopy of a smear of bone marrow, spleen or lymph node, or culture positive. Detection of the organism using specific molecular biological tests can also be used as the basis for etiological diagnosis. The diagnostic criteria are applied as follows: (1) + (2), suspected diagnosis of VL; (1) + (2) + (3), clinical diagnosis of VL; (1) + (2) + (4), definitive diagnosis of VL.

Anti-leishmaniasis drugs, including antimony and amphotericin B, are the only effective treatment for VL. Antimony is the drug of first choice in China because of its low cost, ready availability, and confirmed effectiveness. However, *Leishmania* may be resistant to antimony. Amphotericin B is also effective, but is highly toxic and is associated with severe adverse reactions. Miltefosine and paromomycin are two alternative drugs. However, the prevalence of drug resistance to both amphotericin B and miltefosine has been gradually increasing *(* van Griensven and Diro, 2019; Ibarra-Meneses et al., 2022 *)*. The patient in our case was diagnosed with VL and HLH on the fifth day after admission, and amphotericin B (5mg/d I.V. 1 ×/Day, gradually increased to 0.5mg/kg per day) and dexamethasone sodium phosphate injection (10mg I.V. 2 ×/Day) were immediately administered. Two days later, amphotericin B was replaced with antimony sodium gluconate (6ml 1/day intramuscular injection for 6 days), and the patient’s condition improved rapidly.

## 4 Conclusions

In conclusion, although VL may be fatal, most patients have a good prognosis with timely diagnosis and treatment. As a molecular biological detection method, mNGS has considerable advantages in the diagnosis of VL; however, further research and clinical observation are needed to determine the sensitivity and specificity of this method. Currently, mNGS is more often used to rule out other causes of infection in cases of fever of unknown origin. The method can identify or exclude the presence of thousands of pathogens within 1–2 days, and with reduced costs, more patients may benefit from its use. Although bone marrow biopsy is invasive, it is safe and widely used in clinical practice, particularly as the gold standard diagnostic tool for leishmaniasis with bone marrow involvement. In cases where *Leishmania* does not invade the bone marrow, it may be missed, or multiple samples may be required. Additionally, timely administration of antimony or amphotericin B after VL diagnosis is important, as most patients rapidly improve when provided with the appropriate treatment.

## Data availability statement

The data presented in the study are deposited in the NGDC repository, accession number PRJCA013743.

## Ethics statement

This study was reviewed and approved by The 980th Hospital of PLA Joint Logistics Support Force. Written informed consent was obtained from the patient, for the publication of any potentially identifiable images or data included in this article.

## Author contributions

XZ wrote the first draft of the manuscript. YL, MZ, and ZW reviewed and edited the manuscript. XF, YW, and LY collected the data. JL nursed and followed up the patient. DZ revised the manuscript. All authors contributed to the article and approved the submitted version.
